# Synthesis of Hydrogels and Their Progress in Environmental Remediation and Antimicrobial Application

**DOI:** 10.3390/gels9010016

**Published:** 2022-12-26

**Authors:** Mengshan Song, Jingfeng Wang, Jiabei He, Dongxiao Kan, Kaiyun Chen, Jialu Lu

**Affiliations:** 1 Advanced Materials Research Central, Northwest Institute for Nonferrous Metal Research, Xi’an 710016, China; 2 Shanghai Key Laboratory of Special Artificial Microstructure Materials and Technology, School of Physics Science and Engineering, Tongji University, Shanghai 200092, China

**Keywords:** hydrogels, synthesis, adsorption, antibacterial properties

## Abstract

As a kind of efficient adsorptive material, hydrogel has a wide application prospect within different fields, owing to its unique 3D network structures composed of polymers. In this paper, different synthetic strategies, crosslinking methods and their corresponding limitations and outstanding contributions of applications in the fields of removing environmental pollutants are reviewed to further provide a prospective view of their applications in water resources sustainability. Furthermore, the applications within the biomedical field, especially in wound dressing, are also reviewed in this paper, mainly due to their unique water retention ability, antibacterial ability, and good biocompatibility. Finally, the development direction of hydrogels in the fields of environmental remediation and biomedicine were summarized and prospected.

## 1. Introduction

Hydrogel is one kind of hydrophilic material with a high interconnection structure and high porosity of a three-dimensional polymer cross-linking network. They are generally insoluble in water but show highly absorptive properties, which can be used to release or capture molecules (i.e., CO_2_) while maintaining a good expansion structure [1]. Based on different ways, such as production methods, physical and chemical properties, cross-linking nature, etc. [2], hydrogel can be subdivided to many classifications. A simple but widely adopted classification is whether hydrogel is produced through artificial synthesis or nature. In the synthetic process, different nano-molecules are doped into the hydrogel network to realize the interaction between nanostructures and the polymer chains, thereby enhancing the properties and enabling new applications for special purposes. Although the poor mechanical properties of natural hydrogels limit some of their applications in industry, their advantages are also obvious, i.e., non-toxicity and good biocompatibility [3]. Therefore, the application and imitation of natural polymer materials have become a hot topic in biomedical fields. On the basis of this, an important topic in biological engineering, except for antimicrobial engineering, is how to improve the elasticity of hydrogels, which could ensure the normal operation in various parts of the human body, especially in cartilage groups [4].

In addition, the contribution of hydrogels to environmental protection is also quite remarkable. Polluted water and soil are highly toxic and permeable due to heavy metal ions (cadmium, copper, and nickel [5,6]), and thus lead to high risks of diseases. The traditional methods to decrease heavy metal ions are adsorption, membrane separation, chemical precipitation, ion exchange, etc. [7,8,9]. The process of membrane separation technology is simple and effective, but the surface of the membrane is prone to deposition, which gradually reduces the separation efficiency. The actual separation is costly and requires the assistance of other technologies [10]. Although chemical precipitation is also a relatively simple process, which can condense pollutants into coarse sediment for filtration, it always produces toxic solid waste due to the use of large amounts of chemicals [11,12]. Compared to these technologies, the adsorption method costs less without secondary pollution [13]. Normally, adsorption can be divided into physical or chemical processes. The former relies on the van der Waals interaction and electrostatic interaction, and the latter depends on treating heavy metal contaminants with ion exchange and π-π conjugation [14,15,16].

In this paper, the rapid development of hydrogels in recent years is reviewed with their basic classification and synthesis. The application progress of hydrogels in environmental remediation and biomedial fields is discussed with the existing challenges. In the last part, we discuss the future development of hydrogels.

## 2. Synthesis and Products

Hydrogels are usually prepared through hydrophilic monomers, which can be designed in the synthetic routes prior to preparation to accommodate specific application properties. Hydrogels can be prepared by using natural or synthetic polymers through crosslinking methods. After comprehensive polymerization and hydrolysis steps, the polymer chains are covalently bonded to a complete 3D network microstructure. This method can ensure the mechanical strength of the hydrogel (fracture energy < 100 J/m^−2^) and prevent it from being dissolved in an aqueous environment [17]. Various synthesis strategies for hydrogels are summarized below.

### 2.1. Physical Crosslinking Synthesis

Physical hydrogels, also known as thermoplastic hydrogels or supramolecular hydrogels, can be viewed as reversible solids with viscosity and elasticity that are suitable for applications at room temperature. The process is known as physical crosslinking due to intermolecular interactions (hydrogen bonding [18], electrostatic attraction [19], ionic bonding [20], polymer entanglement [21], crystallite formation and van der Waals forces, etc. [22]). Physical crosslinking processes are mild and easy to prepare, which has lower toxicity and higher biocompatibility than chemical crosslinking processes because it avoids the addition of external chemical crosslinking agents. At the same time, the state of hydrogels prepared by physical crosslinking is unstable, which may lead to the damage of 3D network at high temperature [5]. This ensures the degradability and polluting-free property of the physical hydrogel. However, precisely due to this specific characteristic, the state of the hydrogel will also be changed or even directly dissolved when the environment changes. Thus, it is not suitable for application in some environments which require long-term service.

#### 2.1.1. Hydrogen Bonding

The light, temperature and pH responses of hydrogels make them very popular in biological tissue engineering [20]. Hydrogen bonds, electrostatic attraction and their combinations are self-assembled crosslinking using non-covalent bonds. They are able to create hydrogels with shear thinning properties with extremely elasticity, especially suitable for 3D bioprinting [23,24]. Due to its rheological properties, stress-relaxation behavior and low shear viscosity, it can be injected directly through a very fine needle or catheter and protect cells from high shear forces [25,26]. Therefore, it has been widely used in cell delivery, scaffolds, and embolic biomaterials [27,28]. However, due to the high gel concentration, hydrogels crosslinked by hydrogen bonds are still difficult for injection, and their mechanical strength is not satisfactory as well because of the low crosslinking density given by hydrogen bonding [29,30].

#### 2.1.2. Crystallite Formation

Crystallite formation is also known as the freeze–thaw method, in which a polymer solution is frozen to at least −15 °C, then rapidly melted at room temperature, and repeated until a 3D gel network is formed [31]. During this process, the incorporation of toxic chemicals is completely avoided, and the characteristics of hydrogel can be adjusted by controlling the initial concentration of polymer solution and the number of freezing cycles [31]. For example, multiple cycles will make the gel network grow more closely and thus improve the mechanical strength [21]. At the same time, the higher the initial concentration of the polymer solution, the stronger the mechanical properties [30]. This is because in the frozen state, the polymer molecular chains will interact with each other. For example, the polyvinyl alcohol (PVA) chain forms hydrogen bonds through hydroxyl groups, and then forms ordered polymer crystals locally. Therefore, in the process of cyclic freezing and thawing, opaque hydrogels with the crystal zone as physical crosslinking points will be formed with the continuous increase in PVA strength.

### 2.2. Chemical Crosslinking Synthesis

Hydrogels obtained by chemical crosslinking are also called thermosetting hydrogels or perpetual hydrogels because of their synthetic mechanism: thermal, chemical, and mechanical stability brought by covalent bonding formation [2]. This process includes crosslinking strategies including co-/graft-polymerization crosslinking, photo crosslinking [32], enzymatic crosslinking [33], radiation crosslinking [34], etc. The advantage of chemical crosslinking is that hydrogels can be designed in advance according to the properties required for multiple applications, and the development in modern technologies enable complex material systems with a wide range of applications. However, the drawbacks are still quite obvious: toxic chemical crosslinking agents may harm human health in biomedical applications.

#### 2.2.1. Radiation Crosslinking

Radiation crosslinking is a relatively green chemical crosslinking method that can be used to prepare hydrogels without the aid of chemical initiators and crosslinkers. Hydrogels prepared by radiation crosslinking seems to be more pure and safer, so they are widely used in biomedical fields. However, the high cost prevents it from being manufactured on a large scale; thus, it can only be used for small-scale laboratory synthesis. The process of radiation crosslinking requires high energy sources (microwaves, gamma rays, and ultraviolet light) for excitation, and water is also necessary because it provides free radicals (e^−^, H^+^_2_O*, H_2_O, ·OH), which is necessary during these whole processes [5]. Therefore, radiation crosslinking is also classified as a kind of radical polymerization. Due to the high penetration and wide radiation area of the high-energy electron beam, the breakdown of water molecules and the crosslinking of the polymers occur at the same time, leading to the intermolecular radical recombination, which makes the conversion process of the polymer into hydrogel faster with a more uniform gel structure [34]. In addition, radiation crosslinking will not destroy the original matrix structures, while maintaining biocompatibility and sterilization in the matrix [35].

#### 2.2.2. Crystallite Formation

Photo-crosslinked hydrogels are also known as photocured hydrogels. Similar to radiation crosslinking, in the preparation process, the hydrophilic polymer aqueous solution is irradiated by ultraviolet or visible light into a gel-curing process. In other words, this method supports the in situ hydrogels formation without the addition of any harmful chemicals. It has the characteristics of fast gelation, high mechanical properties, and the manufacture of transparent hydrogels for cell observation [36]. The gelation period is adjustable and inversely proportional to the precursor concentration [37]. So far, photo-crosslinked hydrogels have been used in a wide range of applications, including drug delivery [38], regenerative scaffolds [39], energy storage devices, etc. [40]. Cui et al. formed a do-tyrosine bonds crosslinked silk fibroin hydrogel by photo-crossing for the cultivation of human articular chondrocytes in the biomedical field. According to the study, this hydrogel has a fast gelation speed (10 s to reach the minimum sol fraction, fully formed within 1 min), high cell density (15 million cells/mL), high cell viability (>80 percent) and stable mechanical properties [41]. This shows the great potential of light-mediated REDOX systems in biomedicine and tissue regeneration. In the field of energy storage applications, the type of hydrogel that can be used in supercapacitors is called hydrogel polymer electrolytes (HPE), which act as electrolytes in the system, where they are more eco-friendly and less flammable than ionic liquids [40]. In this process, the HPE will rapidly transport ions during charging and discharging, which will require high electrical conductivity and mechanical properties, as well as reduced resistance. Photo-crosslinking seems to be the best option due to the ultra-fast gelation times (several minutes) and guaranteed properties, including no by-product output [42]. If ultraviolet light is used during this process, the low energy consumption of polymerization at low temperature ensures that the whole process is more environmentally friendly [43].

#### 2.2.3. Enzymatic Crosslinking

Injectable (or spray) hydrogels formed by enzymatic crosslinking in situ reactions have been widely applied in biological tissue engineering. Compared with other crosslinking methods, enzymatic crosslinking has mild reaction conditions and processes, with fast gelation speed and high controllability [44]. More importantly, a nontoxic chemical catalyst is involved in the whole processes, therefore ensuring biocompatibility [45]. Nowadays, it is used for in situ hemostasis or to prevent postoperative adhesions. The materials used include H_2_O_2_, horseradish peroxidase (HRP), transglutaminases, tyrosinase, lysyl oxidase, and phosphatases [46,47].

**Figure 1:**
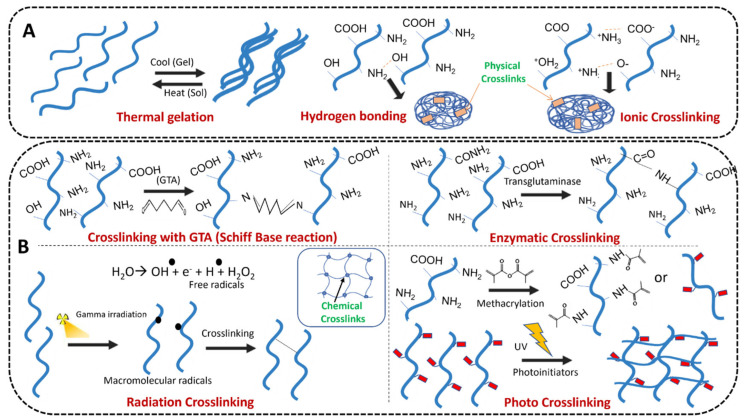
(**A**) Physical and (**B**) chemical crosslinking mechanisms in gelatin hydrogels [2], with the permission of Elsevier.

So far, two of the most basic gelation mechanisms have been described. The physical crosslinking mechanism can be attributed to −NH, −OH or −COOH interactions between polymer chains through hydrogen bonding and ion interactions. The mechanism of chemical crosslinking is to stabilize the hydrogel network by forming 3D chemical covalent bonds. Figure 1 shows the mechanism of physical and chemical crosslinking in gelatin hydrogels. Figure 1A represents three kinds of physical crosslinking mechanisms: freeze–thaw, hydrogen bond and ion interaction [2]. Figure 1B shows four kinds of chemical crosslinking mechanisms: Schiff–Base reaction of complementary groups, enzymatic reaction, high-energy radiation reaction and photocatalytic reaction for free radical polymerization.

### 2.3. Composite Hyrogel Products

As mentioned above, the hydrogels can be simply divided into artificial and natural. Artificial hydrogels are designed according to application requirements with adjustable structures and properties. Therefore, they are also called composite hydrogels. Whether in biomedical or environmental fields, the mechanical properties of hydrogels are the most important issues to be considered in practical applications because they directly relate to the service time in the application processes. Especially when they need to simulate and replace the performance of human soft tissues, fracture or low-adhesion properties may cause secondary injury to the wound, so it is necessary to improve their adhesion [48]. Spray-on hydrogels for emergency wound treatment also require hydrogels to be electrically conductive [49]. After forming a protective layer, hydrogels can release ions to promote cell proliferation and angiogenesis [50]. In addition, the design of hydrogels with high biodegradability and biocompatibility has great advantages in the treatment of heavy metal ions. With the development of science and technology, new composite hydrogels, including nanocomposite hydrogels, interpenetrating polymer networks (IPN and semi-IPN hydrogels), co-polymeric hydrogels, etc., have gradually appeared in the public view.

#### 2.3.1. Nanocomposite Hydrogels

**Figure 2:**
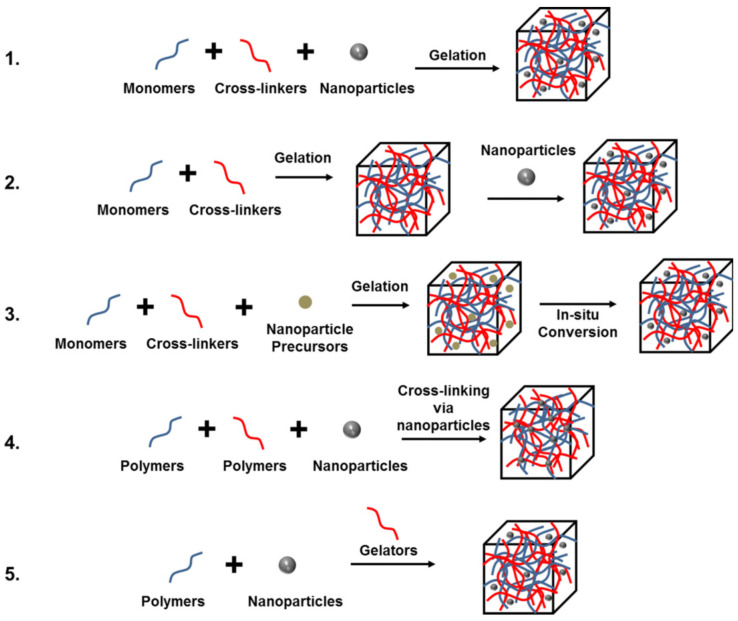
Different ways of doping nanoparticles in hydrogel structures [51], with the permission of the Wiley Online Library.

In order to promote the defects associated with hydrogels or meet the requirements of their related applications, nanoparticles (metal, inorganic, polymer, and carbon-based) are doped into different hydrogel substrates to produce enhanced nanocomposite hydrogels. The doping methods are as follows, as shown in Figure 2: (1) directly form hydrogels from the nanoparticle suspensions; (2) nanoparticles is physically embedded in the hydrogel after gelation; (3) forming nanoparticles in the precursors; (4) using nanoparticles for cross-linking; and (5) using nanoparticles to cross-link with polymers and other gel molecules [51].

In order to clarify the types, properties, and application prospects of various nanocomposite aerogel, here we summarize them in Table 1:

#### 2.3.2. Interpenetrating Polymer Network Hydrogels (IPNs) and Semi-Interpenetrating Polymer Network Hydrogels (IPNs)

Interpenetrating polymer network (IPN) hydrogels are a special class of polymer blends formed by the interpenetrating entanglement of two or more polymers through the network. There is no covalent bond among them, and thus they cannot separate from each other unless the chemical bonds breaks [71]. This kind of unique force will lead to polymers with very different properties or different function binding stabilities so as to realize the complementary properties between binding components. There are two types of IPNs: full IPN and semi-IPN. The full IPN includes two crosslinking polymer networks, and semi-IPN only includes one crosslinking polymer network. Since the 20th century, IPNs have been developed to improve the mechanical properties of hydrogels, and to date, applications including mimicking extracellular matrices and cell culture scaffolds have been gradually developed [72]. Normally, they are formed by building one network first and then forming a second network through external stimuli (light, heat, or chemicals), for which two of the steps do not occur together. The first rigid crosslinking network with certain mechanical properties is formed, and then the network with a low crosslinking degree fills the gaps, as well as dispersing the external stress.

**Figure 3:**
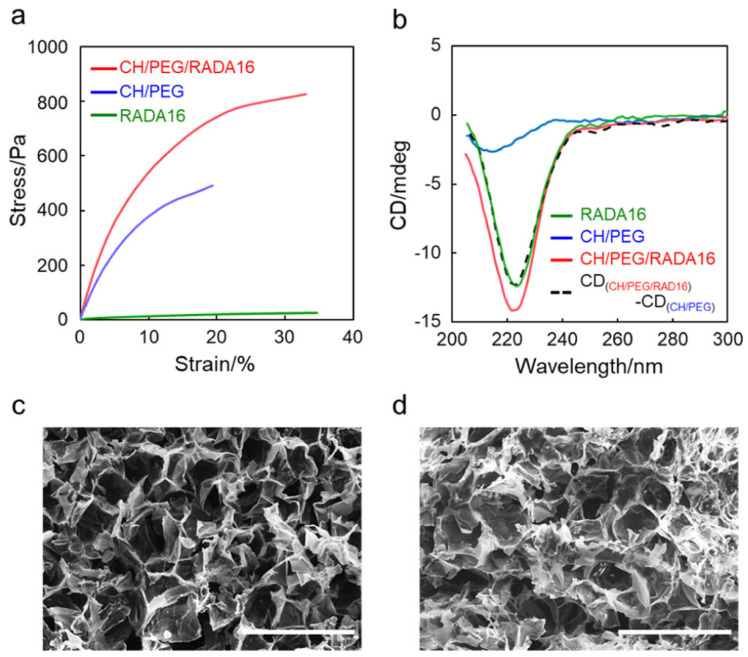
(**a**) Strain-stress curves; (**b**) CD spectra and (**c**,**d**) SEM of CH/PEG, RADA 16 and CH/PEG/RADA 16 crosslinking networks [73], with the permission of ACS Publications.

Interestingly, Hidenori Otsuka et al. designed a one-pot in situ gelation system to construct IPN networks [73]. Two gel networks were formed: RADA16 peptide self-assembly and covalent bonding between chitosan (CH) and N-hydroxysuccinimide ester-terminated poly (ethylene glycol) (NHS-PEG-NHS). Among them, the RADA16 peptide network is formed independently, and the CH/PEG network is formed to attach to the former. Figure 3a shows the stress–strain curves of the IPN hydrogel, RADA16 peptide and CH/PEG network [73]. The results show that the IPN network can effectively improve the mechanical properties of the material networks. Figure 3c,d show that the IPN network structure is not very different from the CH/PEG network, but the network is slightly more compact. At the same time, clinical trials suggest that implantation of the hydrogel could effectively inhibit the inflammatory response in mice and promote protein production, which indicates the feasibility of its application as a scaffold for cell culture.

#### 2.3.3. Double Network Hydrogels (DNs)

Double network hydrogel is an interpenetrating network composed of two materials with different properties, which is similar to IPNs and can even be regarded as a special case of IPNs. They are formed by two independent crosslinking networks. The first one is rigid and brittle, which provides the strength modulus and maintains the structural stress of the network. The second one is soft and ductile, and thus it can withstand and absorb deformation energy when the hydrogel structure is almost broken. DN hydrogels are broadly classified into three types: hybrid hydrogels (physical crosslinking first network, covalent bond second network), physical hydrogels (dual physical crosslinking network), and covalent hydrogels (double covalent bond network). Since the PAMPS/PAAm DN hydrogel was first proposed by Gong et al. in 2003 by using a two-step radical polymerization method, the association between the “sacrificial bonds” and the enhancement mechanism was found and confirmed [74]. DN hydrogels therefore have attracted extensive attention due to their outstanding mechanical properties, such as high Young’s modulus, compressive strain (95 percent), tensile strain (2000 percent), tensile stress (10 MPa), etc. [75]. And the types of DN hydrogels and their properites with corresponding applications are listed in Table 2.

## 3. Application Progress of Hydrogels in Environmental Remediation (Water Sustainability/Protection)

With the progress of civilization and the development of technology, the environmental pollution and threat to human health caused by the discharge of industrial wastewater have gradually attracted the attention of all walks of life. The heavy metals in industrial effluents are toxic and non-biodegradable. They are easily accumulated in organisms, leading to a high incidence of various diseases. Heavy metals of particular concern when dealing with this condition include lead, zinc, copper, nickel, mercury, and chromium [89], which can cause serious health problems in excess, including skin irritation, kidney damage, central nervous system damage, cancer, etc. [90,91,92]. In addition, organic pollutants produced by industrial activities, such as organic dyes (methyl red, methylene blue, rhodamine B, etc.) [93] and nitro compounds [94] have caused great damage to water resources and ecosystems due to their toxicity, low reactivity, high volatility, and high chemical stability. Organic micropollutants (OMPs), which include industrial chemicals, additives, personal care products (PCPs), petroleum derivatives and other substances, are present in low percentages in nature [95]. However, they are still a non-negligible source of water pollution because they are toxic enough to cause immune system dysfunction. A final emerging category of water contaminants is pharmaceutical contaminants, typically the development of bioactive pharmaceutical ingredients (APIs) or pharmaceutical products. Routes of contamination include the direct discharge of unprocessed drugs and the leakage of indigestible parts into the environment through excreta. These drug structures cannot be degraded and cannot be completely captured by conventional treatment techniques [96]. Therefore, the removal of these pollutants is urgent and a controversial challenge for researchers.

**Figure 4:**
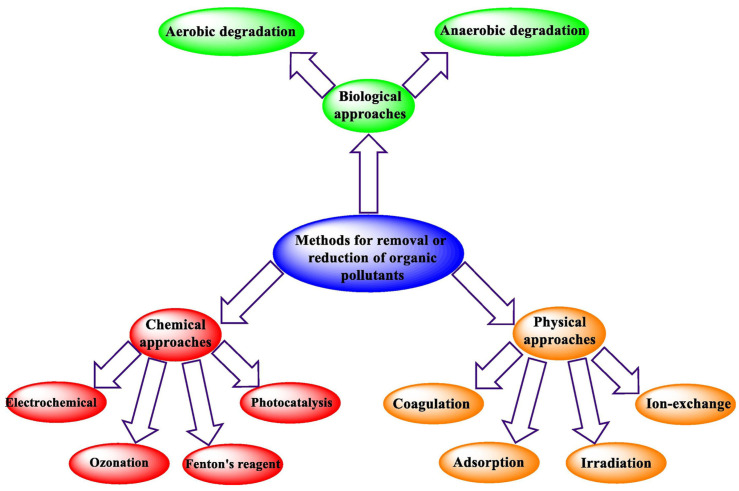
Various strategies of removing organic pollutants [100], with the permission of Elsevier.

The methods for removing organic matter can be roughly divided into three categories: physical, chemical, and biological, including ion exchange, precipitation, adsorption, photocatalysis, aerobic/anaerobic degradation, etc.; various adsorption strategies are shown in Figure 4 [97,98,99,100]. Among them, ion exchange and photocatalysis are relatively mature and step-independent technologies, but considering the costs, adsorption and photocatalysis are being widely used now. There are various absorbents (activated carbon, CLAY, polymer, etc.), but the most interesting ones are those that are renewable and degradable, low-cost, and environmentally friendly absorbents, such as biopolymers—polysaccharide-based hydrogel adsorbents [2]. They have excellent performance in the purification (reduction, degradation, and adsorption) of heavy metal and dye pollution in water resources. Among them, −NH_2_ and −COOH groups play a key role, and their adsorption capacity is also affected by the binding site and porosity of hydrogel.

### 3.1. Removal of Heavy Metals, Dyes, and Organic Pollutants from Wastewater

Due to the various advantages mentioned above, bio-hydrogels have been extensively studied in the aspect of pollutant adsorption. Recently, researchers have committed to maximizing the adsorption capacity of the original hydrogel matrix by adding or modifying functional groups, to obtain bio-based composite hydrogels or functionally modified hydrogels with excellent decontamination quality. Functional groups and substances that can be modified and added include N, O, S, carbon-based materials (GO), nanoparticles, etc. The doping of functional groups can make the design of hydrogels more flexible and is normally used to regulate their structure and properties, including tensile ability, chemical and physical stability, mechanical properties, etc.

The unique 3D structure of the hydrogel ensures the uniform dispersion of metal ions in the interior, promotes swelling, and ensures the sustainability of the gel harvested in the reversible process of adsorption and desorption [101]. At the same time, the support provided by bound water in the structure not only improves the mechanical properties, but also provides nano-transport channels for the diffusion of small molecules, and increases the active sites through molecular interaction, thus leading to the adsorption and fixation of more metal ions [5]. This adsorption process is more similar to ion exchange—the adsorption of heavy metal ions through electrostatic interactions [102]. The physical and chemical interaction of the original polar functional groups (carboxyl group, hydroxyl group, etc.) in hydrogels can ensure the water content of the structure, so as to enhance the adsorption capacity of pollutants. Adhesiveness is also a condition that people need to consider.

However, whether immersed in water or exposed to air, it is certain that hydrogels prefer to contact hydrophilic surfaces rather than interact with hydrophobic surfaces [103].

#### 3.1.1. Functionalized Hydrogel by N, O, S-Containing Groups

N-containing functional groups, such as amines and amidoxime, are some of the most typical and efficient adsorbents. They can be prepared by grafting amine-containing compounds, such as PEI, EDA, DETA, etc., onto the surface of hydrogels. The degree of grafting can be flexibly controlled by temperature, molecular weight, molar ratio, and reaction time. In the adsorption process, N will provide a single pair of electrons as the active site, especially under acidic conditions, through the physical adsorption process to adsorb the positively charged pollutants [104]. Liu et al. prepared SPI/PEI hydrogel by chemical crosslinking, which showed high selective adsorption of Cu (II) in aqueous environment with a variety of heavy metal ions (adsorption capacity increased from 33.5 mgg^−1^ to 136.2 mgg^−1^), especially the selection coefficient of Cu (II)/Zn (II) even reached 250. In addition, the material demonstrated excellent cycle stability and reusability, remaining effective after 5 cycles of desorption [105]. Lu et al. obtained spherical PEI/ chitosan (CS) magnetic beads to eliminate diclofenac sodium (DS), a contaminant of emerging pharmaceutical and personal care products (PPCPs), by using glutaraldehyde (GA)/ epichlorohydrin (ECH) as cross-linkers. The DS adsorption efficiency of the obtained material in 50 mg/L solution is nearly 100 percent, which is 253.32 mgg^−1^. The authors believe that the addition of PEI provides more adsorption sites and improves certain mechanical properties [106]. Godiya et al. prepared a sodium alginate (SA) crosslinked PEI hydrogel. The material showed excellent adsorption toward cationic dye methyl blue (MB), with the removal rate of almost 99 percent in the initial concentration of 100 mg/L aqueous solution, and the adsorption capacity of up to 400 mgg^−1^. At the same time, this method is economic and environmentally friendly; thus, SA/PEI has great potential as a material to adsorb MB from sewage [107]. In general, it can be seen that hydrogels that are functionalized with N-containing groups have significant potential for absorbing metal ions, dyes or new medical pollutants.

Similar to hydrogels functionalized with group N, the grafting of group O in hydrogels also aims to increase the active sites available for adsorption and bonding with pollutants, while the S group has been widely studied because of its remarkable affinity with other components. Functional groups available for grafting include -OH, -COOH, -C=O, -SH, etc. Borsagli et al. designed and prepared a N-acyl thiolated chitosan hydrogel 3D scaffold made from 11-mercaptoundecanoic acid. The adsorption capacity of this material for cationic methyl orange (MO) in water reached 434.89 mgg^−1^ with a removal rate of >80 percent and had antibacterial activity against Pseudomonas aeruginosa. They suggest that the adsorption mechanism is dominated by the affinity of the sulfhydryl group to MO in the hydrogel [108].

#### 3.1.2. Composite Hydrogels

In recent years, the types of composite hydrogels have gradually increased, such as metal NP-modified hydrogels, carbon modified hydrogels, inorganic nano-modified hydrogels, organic polymer modified hydrogels, etc. These modification methods can be used on hydrogels, including biological, inorganic or organic substrates to regulate their physical and chemical properties. Chaudhary et al. synthesized methylene methacrylate/graphite hydrogel composites—a carbon-modified gelatin-based hydrogel—using a relatively green microwave-assisted synthesis method. The results show that the adsorption efficiency of the material for methyl violet (MV) removal is 99.9 percent within 40 min (up to 250 mgg^−1^), and the adsorption efficiency still remains at 95.8 percent after six consecutive cycles of adsorption and desorption [109]. Lone et al. studied a natural polymer double bio-based crosslinking hydrogel, gelatin–chitosan hydrogel, in order to remove heavy metal ions from water. The material exhibits obvious swelling/deswelling characteristics and realizes shape memory. Among many metal ions (lead (II), cadmium (II) and chromium (III)), Hg (II) ions display highly selective adsorption, with an adsorption efficiency up to 97 percent [110]. Dil et al. prepared gelatin-silver/AcA (NPGESNC-AcA) nanocomposite hydrogel by free radical copolymerization and evaluated its ability to remove copper ions from sewage. The results showed that when the pH value is close to 5.5, the maximum adsorption capacity can reach 147.1 mgg^−1^ within 40 min, and the adsorption efficiency remains at 78.7 percent after five cycles of adsorption/desorption. In addition, due to the involvement of silver ions, the hydrogel was endowed with certain antibacterial activity, showing signs of significantly inhibiting the growth of Escherichia coli and Staphylococcus aureus [111].

### 3.2. Water Sustainability

In the environmental applications of hydrogel, the removal of pollutants is one of the most discussed and concerned directions. Beyond that, the collection, storage and utilization of water resources is also an issue that is worthy of discussion and development, for example, water collection from the atmosphere, seawater desalination, agriculture water retention and slow-release hydrogel.

#### 3.2.1. Atmospheric Water Harvesting

**Figure 5:**
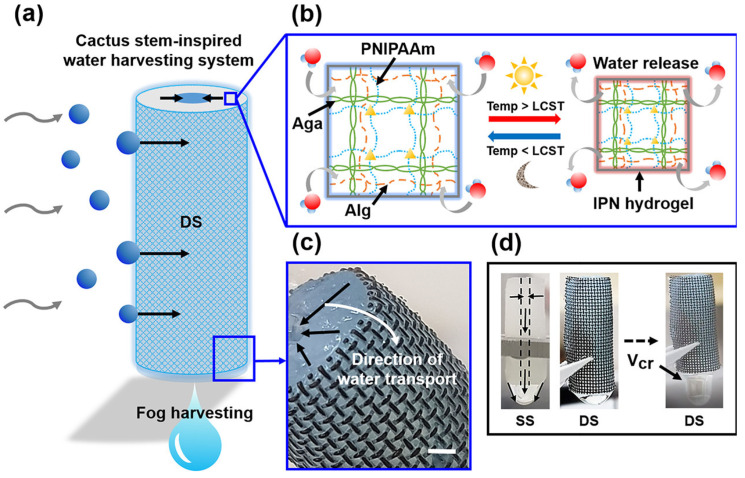
(**a**) Thermoresponsive IPN hydrogel and superhydrophobic copper mesh double structural system; (**b**) Capturing mechanism of IPN hydrogel composed of three gel networks with mimicking the water absorption and long-term storage of cactus stems; (**c**) Directional water transport and storage; (**d**) Schematic diagrams of double structural and single structural systems [115], with the permission of ACS Publications.

Sources of atmospheric water available for harvesting include clouds, fog, and airborne water vapor, which are potentially fresh water in nature. Fog harvesting is considered one of the main means of obtaining fresh water in arid areas. However, these early ways of collecting water have several disadvantages: First, since fog is a suspended micron-scale molecule, collecting it must meet the conditions of saturated humidity. However, not all the water vapor formation requires 100 percent relative humidity, so the process relies on additional power systems that are extremely costly and labor-intensive [112]. The emergence of desiccants has improved this situation, but because of the low water absorption rate, high energy consumption, desiccants still cannot be used as a good water capture material [113]. Therefore, in order to effectively catch fog, humans look to nature for inspiration. The way cactus captures fog benefits from the wetting properties of its stems and the Laplace pressure difference of its spines [114]. Based on these properties, Lee et al. designed and prepared a thermal response fog collection material consisting of a super-hydrophilic interpreting polymer network (IPN) hydrogel and a super-hydrophobic copper mesh (SHPM) [115]. The capture mechanism is shown in Figure 5, the material composed of the IPN and SHPM bilayer structure. IPN is interwoven with agarose helix (Aga), PNIPAAm and calcium alginate (Alg) networks to effectively maintain its water absorption efficiency. Among them, Aga provides rigidity and mechanical properties for the network, Alg maintains the hydrophilicity of the network and then prevents the loss of water molecules, PNIPAAm performs hydrophilic–hydrophobic interconversion near the critical lower solution temperature (LCST); in order to control the release of water molecules, SHPM expands the original specific surface area of the network due to the uneven surface. This increases the rate of fog collection and prevents water molecules from escaping as well.The material exhibits excellent water retention properties and can automatically release the collected water at 34 °C. Its maximum collection efficiency can reach 209 mgcm^−2^h^−1^, maintaining good cycle stability. The collection efficiency can also be improved with the change of wind speed, fiber diameter and the size of fog molecules.

#### 3.2.2. Agriculture Applications

**Figure 6:**
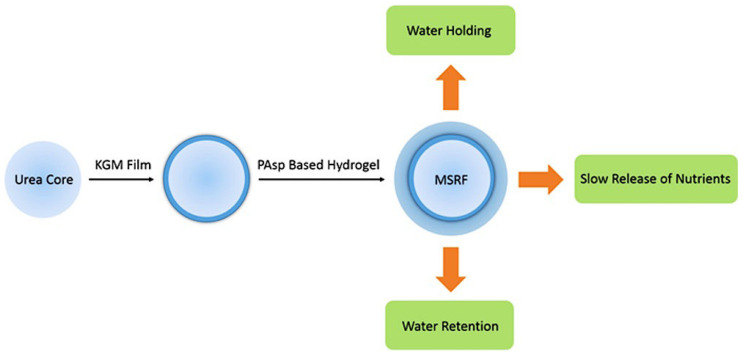
Schematic diagram of new environmental smart fertilizer [118], with the permission of ACS Publications.

In many areas, water shortages and unequal distribution are direct results of drought caused by geography. In this case, the growth of plants and crops showed great problems. To deal with them, the excessive use of chemical fertilizers leads to groundwater pollution and lake eutrophication, but the actual use of chemical fertilizers in crops is only 30–40 percent [116]. Therefore, the development and utilization of new hydrogel fertilizers are very important for agricultural production. Compared with other porous materials, such as sponges, hydrogels with high water absorption and storage capacity have the advantages of improving the utilization rate and delaying soil degradation when they are used as new fertilizers [117]. Slow-release fertilizer (SRF), for example, is an excellent choice for the next generation of fertilizers because it can effectively delay the release of chemicals and avoid the problems caused by excessive fertilizer application. Lu et al. synthesized poly(aspartic acid) and degradable macromolecular crosslinking agent based on L-aspartic acid and put them into the fertilizer to form a new environmental protection fertilizer, as shown in Figure 6: the outer layer is superabsorbent hydrogel, and the lining is urea coated with a deacetylated KGM membrane for the internal coating material [118]. It can achieve effective water absorbing and holding, as well as the effect of the slow release of nutrients. After 23 days of practical application, the maximum water content remained at 22.6 percent, and the microbial degradation was 47.8 wt%.

## 4. Antimicrobial Hydrogel Application (Wound Healing)

### 4.1. Wound Healing Mechanism with Appropriate Dressing Materials

**Figure 7:**
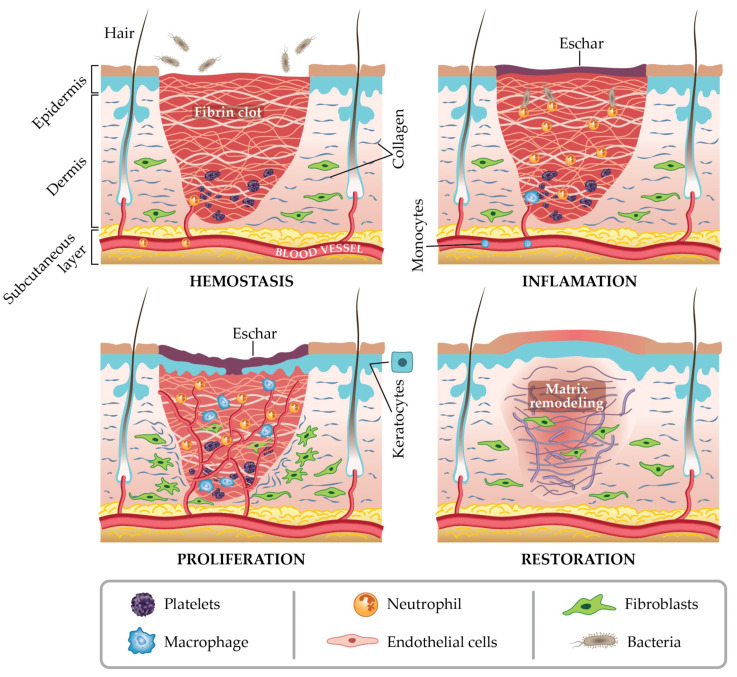
Acute wound healing mechanisms with 4 stages [123], with the permission of MDPI.

Wound healing is a critical area in the biomedical application of hydrogels, because the bacterial infections and drug resistance involved in this process limit the clinical use of many drugs and the efficiency of wound cure. According to the report, about 2 percent of the population in the United States will suffer from health problems, or even death, caused by wounds that will not heal or become infected [119]. A wound is defined as a place where living skin or tissue is damaged or broken. The mechanism of wound healing and infection needs to be understood before selecting the appropriate material. Wound healing can usually be attributed to four stages: hemostasis, inflammation, proliferation, and remodeling [120]. These four phases do not occur independently; they overlap, but the order of occurrence does not change. In the hemostatic phase, platelets begin to accumulate at the wound site to provide a clotting response, while blood vessels contract to cooperatively prevent bleeding. In the inflammatory phase, lymphocytes, neutrophils, and monocytes cooperate to remove foreign materials, bacteria, and some dead and useless cells. In the proliferative stage, blood vessels are formed gradually, and epithelial cells migrate from the wound site to achieve re-epithelialization. During the remodeling phase, the tensile strength of the wound increases, gradually reaching 80 percent of its pre-injury strength [121]. It is important to note that these processes usually occur in acute wounds; they normally will heal within 8–12 weeks, depending on the size and depth of the wound [122]. Figure 7 shows the mechanism of acute wound healing processes. For chronic wounds, such as ulcers, the long inflammatory period caused by persistent infection prevents them from healing properly in a short period of time [123].

Actually, there are many other types of wound dressings on the market, aside from hydrogels, that we would pay attention to, such as hydrocolloids, foam dressings, film dressings and traditional dry dressings. Traditional dry dressings are used in the earliest stages of the wound for acute hemostasis and emergency treatment, but they are not beneficial for wound healing. Dry dressings tend to stick to the wound and cause secondary damage because they do not maintain a moist environment around it [124]. Hydrocolloids are composed of a hydrophilic/colloids layer containing proteins or polysaccharides and a backing material containing non-woven polyester fibers. They have semi-absorbent properties to water and oxygen, creating a moist wound environment by absorbing wound fluid; therefore, it is more suitable for chronic wounds with small or moderate exudate. Hydrocolloids come in a variety of shapes: powder, paste or flake dressings can be used to treat wounds of varying depth and breadth. However, the defect is that it is unable to treat necrotic wounds with excessive exudation. The hypoxia and excessive dampness caused by such an environment are not conducive to healing. In addition, hydrocolloids may generate a distinct odor at the wound site. Foam dressings consist of hydrophilic polyurethane foam and are commonly used as an alternative to hydrocolloids. The absorbent properties make them very effective in wounds with a lot of exudates. Foam dressing is very conformable to skin surfaces, and can be made to the right size according to the shape of the wound, which plays a strong role in protecting the wound sites. These properties above allow it to remain on the wound for up to seven days, effectively reducing secondary damage caused by dressing stripping and replacement [125]. However, foam dressings are not suitable for wounds with just too little exudation, which may result in a dry wound environment that is not conducive to recovery. Film dressings are transparent materials made of polyurethane that are waterproof, skin adhesive, and semi-permeable to oxygen and water vapor. They are only suitable for wounds where the surface of the skin is very shallow and there is very little exudate because they cannot absorb the exudate. Although thin film dressings are also replaced infrequently (4–5 days), their impenetrable nature limits the applications at wound sites [126].

By contrast, hydrogels have advantages over the above materials in that they are flexible, have certain mechanical properties, and can cover different types of wounds. At the same time, they have high permeability to oxygen, water and metabolites. Hydrogels are colorless and odorless, promote the autolysis of dead tissue to avoid dryness, and can be loaded with antibacterial agents and active wound-healing agents to promote wound healing. They are also able to respond to various stimuli and are able to being crosslinked in situ. Therefore, we believe that hydrogel is one of the most suitable and promising materials for wound dressing compared to other materials.

### 4.2. Hydrogel Strategies for Acute Wounds

New external drugs need to be developed to maintain moisture in the wound, while isolating bacteria and promoting healing. Among the various of materials, a well-combined hydrogel should have all the advantages of a topical wound application (dressing): (1) it keeps the wound moist, retains the wound exudate but does not cause obvious damage, and the cold and damp environment reduces pain as well; (2) biocompatible, does not cause other reactions; (3) transparent material to monitor wound recovery; (4) no secondary damage when uncovered; and (5) excellent oxygen permeability [125,127]. Therefore, antibacterial hydrogels and their complexes have gradually replaced traditional wound dressings as a new generation of choice. The antibacterial properties of hydrogels are mainly manifested in two ways: the inherent antibacterial activity of the material itself and the additional antibacterial activity obtained by doping or crosslinking. Because of the highly interconnected 3D structure, antimicrobial molecules can be delivered to the vicinity of the wound through the gel network, and the dose of antimicrobial agents can be controlled, reducing resistance while controlling infection [128,129].

Inherent antibacterial hydrogels refer to those hydrogel materials that come from nature and have certain antibacterial properties. People usually pay attention to polysaccharide bio hydrogels constructed based on natural polymers, because they can be easily obtained from various sources, such as animals, plants, algae and microorganisms. They are safe and non-toxic, with good biocompatibility, renewability and biodegradability, for example, chitin and chondroitin sulfate in living organisms; cellulose and carrageenan from plant cell walls and algae, etc. [130]. These natural polymers are usually cationic. They interact with bacterial cell membranes to reduce bacterial resistance and activity and are one of the effective ways to combat multidrug-resistant bacteria [129].

Based on natural antibacterial hydrogel network, physical doping or the chemical crosslinking of other structures or substances can control the physical and chemical properties of materials to achieve different application purposes. Nano-metal particles are one of the high-potential substances to be doped into hydrogel structures because of their small size and high antibacterial activity [131]. Silver ion is one of the earliest wound treatment materials used because of its antibacterial properties and biocompatibility, even dating back to B.C. In the field of modern medicine, silver ions exist as an alternative against antibiotic-resistant bacteria. It can be doped in a variety of ways in different hydrogel substrates in order to control the release rate and particle size of silver in the body for use against different wounds. However, most of the current studies use the chemical reduction method to dope nano-metal particles, in which the chemical reagents used will cause different degrees of poisoning of organisms. Therefore, it is important to develop new doping methods in this field to achieve the goal of reducing biohazards. Jube et al. investigated an in situ synthesis of silver NPs-doped polyvinyl alcohol/gum acacia (PVA-GA) hydrogel by using gamma ray radiation-induced crosslinking [132]. The particle size of silver ions ranges from 10 to 40 nm, and its loading increases with the increase in structure cross-linking density. The material showed good biocompatibility, thermal stability and antibacterial activity, especially against Gram-negative bacterium, Escherichia coli. At the same time, the higher the concentration of GA, the smaller the particle size of the silver ions loaded in it, and the release rate in vivo also increases accordingly. Other metal nanoparticles (gold and copper) and metal oxides (zinc oxide, copper oxide, and titanium dioxide) are less well-studied than silver ions, but they still have their own characteristics and application scenarios. Gold ions have low cytotoxicity, high antioxidant capacity, and unique electronic and optical properties; their compounds show antibacterial activity against most pathogens growing at the wound site. It makes cell contents exudate by contacting the cell membrane, inhibiting protein synthesis, therefore achieving a bactericidal effect [133]. Mahmoud et al. studied the effect of gold ions on wound healing in rats by doping gold ions into a poloxamer 407 hydrogel matrix [134]. The material showed good stability and high antibacterial activity (*S. aureus* and *P. aeruginosa*). It can enhance the skin re-epithelialization process and collagen formation, and achieve the complete healing of daily wounds within 14 days. However, they point out that gold ions are not widely used because of their high cost, and lack of theoretical studies on their size, surface chemistry and detailed wound-healing mechanisms.

### 4.3. Hydrogel Strategies for Chronic Wounds

**Figure 8:**
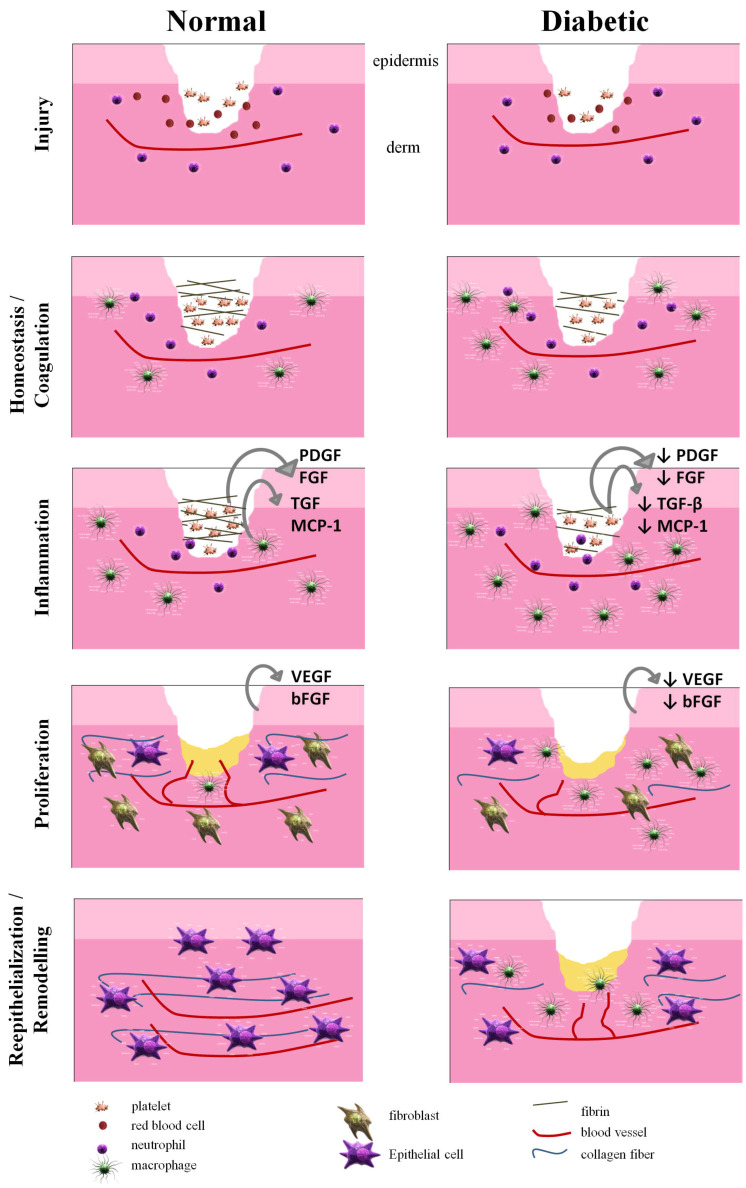
Mechanisms of acute and chronic wound healing [125], with the permission of Elsevier.

With the rise of diabetes patients on worldwide, more and more people are suffering from chronic and non-healing wounds caused by inflammation and lack of oxygen [135]. However, there are not as many treatments for chronic wound studies as there are for acute wounds [136]. Figure 8 compares the healing process of chronic (diabetic wounds) and acute wounds [125]. Usually, the healing mechanism of chronic wounds does not completely follow the general wound healing processes (hemostasis, inflammation, proliferation, and restoration) because of complex conditions induced by hypoxia, lack of blood supply, lack of nutrients, hypothermia, impaired granulocyte, chemotactic and macrophage function, impaired expression of growth and angiogenic factors, potential infections, etc. Therefore, conventional hydrogel dressings cannot meet the need to combat recurrent inflammation; thus, some natural antibacterial hydrogels, as well as hydrogels loaded with antibiotics in the substrate, have become one of the main strategies to combat these chronic wounds. Like the treatment of acute wounds, natural inherent antibacterial hydrogels are also suitable for the treatment of chronic wounds. They are usually a bio-matrix, which works like human skin cells and can mimic their working environment, but have low mechanical properties, unstable sources and defects that may lead to the spread of infectious diseases. So, subsequent modification is also applicable. Hoque et al. selected vancomycin as an antibiotic loaded onto a natural antimicrobial hydrogel (N-(2-hydroxy) propyl-3-trimethylammonium ammonium chloride, HTCC) and a viscous biopolymer (polydextran aldehyded, PDA) composite matrix [137]. The antibiotic is released in a pH-dependent manner and has long antibacterial activity (MRSA and methicillin-resistant Staphylococcus aureus) due to the dual properties of antibiotic release and self-exposure against bacteria. It is not toxic to human tissues.

### 4.4. Other Wounds

Other types of wounds, such as acute wounds due to intraoperative arterial rupture, would need special treatments. Because intraoperative bleeding is usually accompanied by high systolic blood pressure, the amount of bleeding is large, and the blood flow is fast, so it is difficult to control. At the same time, postoperative adhesion is also one of the serious problems [138]. Therefore, enzymatic responsive hydrogels are expected to be one of the options for the emergency management of intraoperative arterial bleeding in addition to hemostatic forceps. Sun et al. reported an enzyme-responsive hydrogel that could be used to stop bleeding in the external luminal artery in less than 10 s [139]. The material has great adhesion property and biocompatibility, supports injection and minimizes adhesion, and is expected to be one of the effective strategies for the management of intraoperative minimally invasive acute hemorrhage. Besides this, Cheng et al. designed and prepared a biodegradable ε-poly-L-lysine loaded N, O-CS/TOCN composite sponge, which can be used as a potential wound dressing to treat postoperative adhesions. Through experiments, it was found that this material can guarantee antibacterial and anti-inflammatory activity in vivo, and promote wound hemostasis and healing [140].

## 5. Conclusions and Perspectives

Hydrogel is a 3D network with a highly interconnected structure. In this article, we reviewed the different synthetic methods and matrix components of hydrogels, and described their applications in environmental remediation and antibacterial—especially in the fields of water resources protection and wound management, as the characteristics of hydrogels can be changed by regulating their internal structure and cross-linking mode. There are a series of sources of matrix and doped/cross-linked functional groups that can be used to change the hydrogel structure, and thus to distinguish the hydrogels. The modified hydrogels have wild applications in industrial and commercial fields due to their porosity, swelling property, hydrophilic/hydrophobic and environmental sensitivity. Then, through these advantages of properties and their relevant mechanism, we can also prospect hydrogels’ potential applications in the future.

From the perspective of water resources treatment, sewage discharge from textile factories is a main source of water resources pollution. This kind of sewage usually contains a large number of heavy metals and harmful chemicals, which cannot be naturally degraded, posing a great threat not only to the environment, but also to the health of organisms through infiltration into groundwater and other ways. Bioinspired hydrogels have gained much attention because of their various functional groups, e.g., hydroxyl and epoxy groups, which have been proved to remove heavy metal ions from sewage effectively. This kind of modified hydrogel is also reusable, environmentally friendly, and has excellent stability, making it an irreplaceable material in the field of sewage purification. From the view of the sustainable development of water resources, hydrogels have the ability to mimic nature organics and plants that can capture water droplets from fog and clouds, which means that they can be used in arid areas. Moreover, their unique swelling and water retention can be applied to the agricultural field, forming a special kind of fertilizer so as to avoid a series of environmental problems caused by excessive fertilization.

Biobased polysugar hydrogels are clearly the most suitable choice for biomedical applications (wound treatment and implantable medical devices) because of their safety and biocompatibility. It has been proven that natural antibacterial hydrogels are suitable for wound treatment. However, due to the presence of drug-resistant bacteria, synthetic composite antibacterial hydrogels are an alternative. The hydrogel framework is an excellent carrier, which can be loaded with antibiotics or nano-metal particles to optimize its performance. Therefore, the future development direction is to design and optimize natural antibacterial hydrogels to make them have the potential to combat more complex wound conditions. In addition, polymer hydrogels have great potential in other biomedical fields, such as drug delivery and heart/bone repair. However, the corresponding problems also include (1) how to ensure the coexistence of dispersibility, hydrophilicity and biocompatibility in vivo; (2) how to carry out large-scale industrial production and ensure suitable pore size of porous materials; (3) how to ensure the mechanical properties of materials and the stability of their services in the body; and (4) how to ensure the degradability and sustainability of materials. In the future, only by overcoming these difficulties will researchers be able to use hydrogels as biomedical materials in vivo safely and effectively [141,142].

In general, people’s attention to hydrogel materials should not stay in a single direction. There should also be a development trend of this material in the future to find the commonality between material properties and realize the integration of resources in different fields, such as environment and biomedicine.

## Figures and Tables

**Table 1:** Types of nanocomposite hydrogels and their properties with corresponding applications.

Types of Nanocomposite Hydrogels		Properties	Applications
Metal Nanoparticle Hydrogels	Silver NPs	Antimicrobial properties Biocompatibility Non-toxic Low mechanical properties Low binding affinity with surfaces	Dental fillings [52] Wound dressing [53] Concentration sensors [53] Functional antibacterial coatings [54] Eye impants [54]
	Gold NPs	Antimicrobial properties Conductivity High costs	Remote drug dilivery [55] Remote control microfluidic valve [56]
	Other NPs	Magnetism properties Low costs High surface/volume ratio	Catalysts [57] Liquid separation [57]
	Metal oxide NPs	Ferromagnetic Semiconductivity Light response	Toxic ion absorber [58] Magnetically driven actuators [59] UV protection [60] Photocatalytic [60]
Carbon Nanoparticle Hydrogels	Carbon Nanotubes	Electrical, thermal- stimulation response High ductility	Photothermal drug dilivery [61] Crosslinking agent [62]
	Graphene	Hydrophilic Conductivity	Site-specific gene delivery [63] Photothermal drug delivery [61]
Polymer-based Nanoparticle Hydrogels	Dendrimers/Hyperbranched polymers	High loading efficiency High mechanical properties	Encapsulate hydrophobic drug molecules [64]
	Liposomes	High elasticity Hydrophilic/hydrophobic within a same structure	Drug delivery [65] Wound dressing [65]
Inorganic-based Nanoparticle Hydrogels	Si-NPs	Mechanical properties Antimicrobial properties	Implantable material [66] Carrier of catalysts or functional materials [67]
	Glass ceramics	Ordered, stable structure	Induction agents for bone growth [68]
	Hydroxyapatite	High rigidity Low biosorption rate Poor stimulating effect on growth of new tissues	Fillers for amputation bone replacement [69] Plant coatings [69]
	Calcium phosphate	Calcium supply	Stimulate bone growth [70]

**Table 2:** Types of DN hydrogels and their properties with corresponding applications.

Types of DN Hydrogels	Reinforcing Mechanism	Properties	Applications
Covalent	Sacrificial bonds	Resistance to biological contamination High strength High toughness Dimensional stability	Hard tissue replacement [76] Bone formation [77] Self-growing materials [78]
Non- covalent	(Hybrid) Metal ion coordination	Complete recovery rate High strength High toughness Convenient control	3D printing Promote skull regeneration Protect brain tissue [79]
	(Hybrid) Hydrogen bonding	Unstable in aqueous environment, stable in hydrophobic environment Biodegradable Non-toxic	Adsorption of heavy metal ions [80] Biomedical applications [81]
	(Hybrid) Hydrophobic interactions	High tensile strength Large fracture strain Good toughness	Biological assembly [82] Synthesis of bioinspired DN hydrogels [83]
	(Hybrid) Host-guest interactions	Injectable Weight-bearing Cytocompatibility Adjustable mechanical properties	Biomedical stents [84]
	(Physical) Metal ion coordination	High affinity Conductivity	Electrical skins Actuators [85]
	(Physical) Hydrogen bonding	Non-cytotoxic Salt tolerance Very low adhesion to tissues	Replacement of living tissue [86]
	(Physical) Hydrophobic interactions	Self-healing	Electronic sensors [87]
	(Physical) Host-guest interactions	Shear thinning Rapid thixotropic behavior	Vivo injection [88]

## Data Availability

Not applicable.
